# Hepatectomy for Hepatocellular Carcinoma in Elderly Patients: Perioperative Outcomes in the Modern Minimally Invasive Era

**DOI:** 10.3390/jcm15072753

**Published:** 2026-04-05

**Authors:** Byeong Gwan Noh, Young Mok Park, Myunghee Yoon, Hyung Il Seo, Myeong Hun Oh, Suk Kim, Seung Baek Hong

**Affiliations:** 1 Division of HBP Surgery and Transplantation, Department of Surgery, Biomedical Research Institute, Pusan National University Hospital, Busan 49241, Republic of Korea; sagerbk@naver.com (B.G.N.); ymh@pusan.ac.kr (M.Y.); ohmyunghun@naver.com (M.H.O.); 2 Department of Surgery, School of Medicine, Pusan National University, Yangsan 50612, Republic of Korea; 3 Department of Radiology, Biomedical Research Institute, Pusan National University Hospital, Busan 49241, Republic of Korea; 4 Department of Radiology, School of Medicine, Pusan National University, Yangsan 50612, Republic of Korea

**Keywords:** carcinoma, hepatocellular, hepatectomy, laparoscopy, aged

## Abstract

**Background:** As life expectancy increases, a growing number of elderly patients are considered for curative hepatectomy for hepatocellular carcinoma (HCC). However, perioperative outcomes in elderly patients in the contemporary era of minimally invasive liver surgery remain incompletely defined. **Methods:** We retrospectively reviewed 277 consecutive patients who underwent elective curative hepatectomy for HCC between 2019 and 2023. Outcomes were compared using age thresholds of ≥75 and ≥80 years. The primary endpoints were 90-day mortality and major postoperative complications (Clavien–Dindo grade ≥ III). Multivariable logistic regression identified predictors of major complications. **Results:** Elderly patients had more comorbidities, whereas liver function, tumor characteristics, and extent of resection were comparable across age groups. Laparoscopic hepatectomy was performed more frequently in patients aged ≥80 years. Major complication rates and 90-day mortality were similar regardless of age, with no deaths among patients aged ≥75 or ≥80 years. Age ≥75 years, higher ALBI score, major comorbidities, and longer Pringle maneuver time were independently associated with major postoperative complications. **Conclusions:** Hepatectomy for hepatocellular carcinoma may be performed with acceptable short-term outcomes in carefully selected elderly patients, including octogenarians. Chronological age alone should not be considered an absolute contraindication to surgery, although findings should be interpreted with caution.

## 1. Introduction

As global life expectancy continues to rise, surgeons increasingly encounter elderly patients with hepatocellular carcinoma (HCC) who may be candidates for curative liver resection [1]. HCC is frequently diagnosed in older individuals, and the proportion of elderly patients undergoing evaluation for surgical treatment continues to increase in routine practice. Hepatectomy remains a potentially curative treatment for HCC in appropriately selected patients. Nevertheless, advanced age has traditionally been considered a risk factor for postoperative morbidity and mortality because elderly patients often have reduced physiologic reserve and greater vulnerability to perioperative stress, particularly in the presence of multiple comorbidities [2,3]. In broader surgical populations, increasing age and higher preoperative risk burden have been associated with greater postoperative morbidity and short-term mortality, emphasizing the need for careful selection and risk stratification in elderly surgical candidates [2,3]. For this reason, the role of age in determining surgical eligibility remains an important clinical issue, especially in patients considered for major abdominal surgery.

Despite these concerns, accumulating evidence suggests that chronological age alone should not automatically preclude hepatectomy. Several cohort studies focusing on elderly patients with HCC have reported acceptable perioperative morbidity and low mortality after liver resection, with outcomes comparable to those of younger patients when patient selection is appropriate [4,5]. These findings have led to increasing recognition that age alone may be an insufficient basis for excluding elderly patients from potentially curative resection. At the same time, however, interpretation of these data is not straightforward. In daily practice, elderly patients who proceed to hepatectomy are often highly selected, and the extent to which favorable outcomes reflect the effect of age itself, underlying physiologic status, or selective surgical indication remains uncertain. This distinction is clinically relevant because it directly influences how surgeons counsel elderly patients and how operative risk is balanced against potential oncologic benefit.

In parallel, perioperative risk assessment has increasingly shifted toward biological and functional determinants of surgical risk. Frailty has emerged as an important predictor of adverse postoperative outcomes in elderly patients and has been incorporated into perioperative guidance for older adults undergoing surgery [6]. Large surgical cohort studies and meta-analyses have demonstrated that frailty is independently associated with increased postoperative complications and mortality across multiple surgical specialties [7,8]. Liver surgery-specific evidence similarly suggests that frailty adversely affects outcomes in patients undergoing oncologic liver resection [9]. In addition, sarcopenia and unfavorable body composition measured on preoperative imaging have been associated with worse short-term outcomes following liver resection, further supporting the clinical importance of functional and nutritional assessment beyond chronological age [10]. These observations suggest that chronological age alone does not adequately capture operative risk in elderly patients and that a more comprehensive assessment of physiologic reserve is required.

At the same time, the growing adoption of minimally invasive liver resection has changed the perioperative landscape in hepatobiliary surgery. Meta-analyses of randomized controlled trials comparing laparoscopic and open liver resection have demonstrated reduced perioperative morbidity and faster postoperative recovery with minimally invasive approaches while maintaining comparable oncologic outcomes in selected patients [11]. Evidence focusing specifically on HCC also suggests that laparoscopic liver resection can provide perioperative advantages without compromising oncologic adequacy compared with open surgery [12,13]. These potential benefits may be particularly relevant in elderly patients, in whom minimizing operative stress, blood loss, pain, and postoperative immobilization may help preserve functional recovery. As minimally invasive techniques become more widely adopted, the question of whether advanced age independently influences perioperative outcomes requires reappraisal within the context of contemporary surgical practice rather than older operative eras.

However, even in the modern era of minimally invasive liver surgery, the independent impact of advanced age on short-term outcomes following hepatectomy for HCC remains incompletely defined. Available data are heterogeneous with respect to patient selection, operative approach, and perioperative management, and relatively few studies have focused specifically on contemporary cohorts. In addition, it remains unclear whether the effect attributed to age reflects chronological aging itself or the combined influence of liver function, comorbidity burden, and selective surgical indication. The present study therefore aimed to evaluate 90-day mortality and major postoperative complications after curative hepatectomy for HCC in elderly patients treated at a tertiary referral center, with particular focus on the influence of chronological age in contemporary surgical practice.

## 2. Patients and Methods

### 2.1. Study Design and Setting

This retrospective cohort study was conducted at Pusan National University Hospital, a tertiary referral center for hepatobiliary surgery. Consecutive patients who underwent elective liver resection for HCC between May 2019 and December 2023 were reviewed. The study was approved by the Institutional Review Board of Pusan National University Hospital, and the requirement for informed consent was waived due to the retrospective nature of the study.

Clinical data were collected from a prospectively maintained institutional hepatobiliary surgery database and supplemented by review of electronic medical records. Because the follow-up duration was relatively limited, the study focused primarily on short-term perioperative outcomes rather than long-term oncologic results.

### 2.2. Patient Selection

During the study period, 277 consecutive patients met the inclusion criteria and were included in the analysis. Patients who underwent emergency surgery, concomitant major extrahepatic procedures, or non-curative resections were excluded in order to minimize confounding related to operative complexity.

Clinical variables including demographic characteristics, comorbidities, preoperative laboratory findings, operative variables, and postoperative outcomes were obtained from a prospectively maintained institutional hepatobiliary surgery database. 

Treatment decisions were made based on tumor characteristics (including size, number, and location), hepatic functional reserve, and patient comorbidities. All patients included in this study had resectable hepatocellular carcinoma and were considered candidates for curative hepatectomy. In our practice, locoregional treatments such as transarterial chemoembolization or ablation are typically reserved for patients with impaired hepatic function, significant operative risk, or tumors not amenable to complete resection.

### 2.3. Age Stratification

To evaluate the effect of advanced age on perioperative outcomes, patients were stratified using two predefined age thresholds commonly used in surgical literature: ≥75 versus <75 years and ≥80 versus <80 years. These thresholds were selected to reflect clinically relevant age groups frequently used to define elderly and very elderly populations in hepatobiliary and general surgical studies.

### 2.4. Surgical Technique

All resections were performed by experienced hepatobiliary surgeons using either an open or minimally invasive approach, including laparoscopic liver resection. The operative approach was determined according to tumor location, hepatic functional reserve, patient condition, and surgeon discretion.

### 2.5. Definition of Liver Resection

The extent of liver resection was classified as minor or major hepatectomy according to the number of Couinaud segments resected. Major hepatectomy was defined as resection of three or more Couinaud segments, including right anterior and right posterior sectionectomies. Minor hepatectomy included all resections involving fewer than three segments. Whenever technically feasible, parenchymal-sparing approaches were preferred, particularly in elderly patients, in order to preserve postoperative liver function.

### 2.6. Outcome Measures

The primary endpoints were 90-day postoperative mortality and major postoperative complications. Major complications were defined as Clavien–Dindo grade ≥ III, representing clinically significant events requiring surgical, endoscopic, or radiologic intervention. Secondary outcomes included overall complication rates, length of hospital stay, intraoperative variables (including operative time, estimated blood loss, and intraoperative transfusion), and postoperative liver function trends. Postoperative liver function was assessed using serial measurements of aspartate aminotransferase, alanine aminotransferase, prothrombin time, and total bilirubin on postoperative days 1, 3, and 5. The incidence of post-hepatectomy liver failure was also evaluated according to the criteria of the International Study Group of Liver Surgery (ISGLS). Outcomes were assessed during the index hospitalization and during outpatient follow-up within 90 days after surgery. Mortality was defined as death from any cause within 90 days after surgery.

### 2.7. Statistical Analysis

Continuous variables are presented as mean ± standard deviation or median (interquartile range), as appropriate. Categorical variables are presented as frequencies and percentages. Comparisons between groups were performed using Student’s *t*-test or the chi-square test as appropriate. Fisher’s exact test was used when expected cell counts were <5. Logistic regression analysis was performed to identify independent predictors of major postoperative complications. Variables considered clinically relevant or demonstrating *p* < 0.05 on univariable analysis were included in the multivariable model. Statistical support was provided by the Department of Biostatistics at the Biomedical Research Institute of Pusan National University Hospital. Analyses were performed using SPSS version 20.0 (IBM Corp., Armonk, NY, USA).

## 3. Results

### 3.1. Baseline Patient Characteristics

Baseline clinicopathologic characteristics according to age are summarized in Table 1. Using the 80-year threshold, patients aged ≥80 years were more frequently female compared with those aged <80 years (37.8% vs. 20.4%, *p* = 0.032) and had a significantly higher prevalence of comorbid conditions (94.6% vs. 37.1%, *p* < 0.001). Other baseline variables, including ASA physical status, prior abdominal surgery, body mass index, and liver function indices such as ALBI and MELD scores, were comparable between groups. Although major hepatectomy tended to be less common in patients aged ≥80 years, the difference did not reach statistical significance. Notably, laparoscopic liver resection was performed more frequently in patients aged ≥80 years than in younger patients (94.6% vs. 80.8%, *p* = 0.040).

Tumor-related characteristics, including tumor size, proportion of tumors ≥5 cm, tumor multiplicity, and microvascular invasion, were generally similar between groups. When stratified using the 75-year threshold, similar trends were observed. Female sex and the burden of comorbidities were both higher in patients aged ≥75 years (both *p* < 0.05). Microvascular invasion was less frequent among patients aged ≥75 years than among those aged <75 years (21.4% vs. 35.3%, *p* = 0.045). Liver functional reserve, extent of hepatectomy, operative approach, and overall tumor burden did not differ significantly.

### 3.2. Operative and Postoperative Outcomes

Operative details and postoperative outcomes according to age are presented in Table 2. Patients aged ≥80 years had shorter operative times compared with those aged <80 years (189.7 ± 61.2 vs. 226.6 ± 74.3 min, *p* = 0.028). Intraoperative transfusion, estimated blood loss, use of the Pringle maneuver, and length of hospital stay were not significantly different between the groups.

Postoperative biochemical parameters, including AST, ALT, prothrombin time, and total bilirubin measured on postoperative days 1, 3, and 5, were broadly comparable. Although AST levels on postoperative day 5 were lower in patients aged ≥80 years (*p* = 0.002), no other clinically meaningful differences in postoperative liver function trends were observed.

Using the 75-year cut-off, operative time remained shorter in patients aged ≥75 years compared with those aged <75 years (203.6 ± 55.4 vs. 229.1 ± 77.1 min, *p* = 0.008), and estimated blood loss was also lower (416.0 ± 257.2 vs. 557.5 ± 707.6 mL, *p* = 0.022). Rates of major postoperative complications (Clavien–Dindo grade ≥III) and 90-day mortality were not significantly different between age groups using either age threshold. Overall 90-day mortality was low, and no deaths occurred among patients aged ≥75 or ≥80 years.

### 3.3. Predictors of Major Postoperative Complications

Multivariable logistic regression analysis was performed to identify independent predictors of major postoperative complications, and the results are summarized in Table 3. Age ≥75 years was associated with major postoperative complications in the adjusted analysis; however, this finding should be interpreted cautiously due to potential residual confounding and limited event numbers (odds ratio [OR], 9.92; 95% confidence interval [CI], 2.86–34.45; *p* = 0.001). Higher ALBI score (OR, 6.57; 95% CI, 1.17–36.81; *p* = 0.032), the presence of comorbidities (OR, 3.85; 95% CI, 1.28–11.58; *p* = 0.017), lower body mass index (BMI) (OR, 0.83; 95% CI, 0.71–0.98; *p* = 0.024), and a higher number of Pringle maneuver cycles (OR, 1.61; 95% CI, 1.16–2.23; *p* = 0.004) were also independently associated with major postoperative complications. Other variables, including surgical approach, MELD score, ASA classification, underlying cirrhosis, previous operation, operative time, estimated blood loss, and intraoperative transfusion, were not significantly associated with major postoperative complications.

## 4. Discussion

### 4.1. Impact of Age on Perioperative Outcomes After Hepatectomy

As populations continue to age, an increasing proportion of patients considered for hepatectomy are elderly and frequently present with multiple comorbidities [1,2]. In this context, clarifying the role of chronological age in perioperative risk remains clinically relevant. In the present study, advanced age showed a nuanced relationship with short-term outcomes after hepatectomy for hepatocellular carcinoma. Patients aged ≥80 years did not experience higher rates of major postoperative morbidity or 90-day mortality compared with younger patients, whereas age ≥75 years was associated with major postoperative complications in the adjusted analysis. Although differences in laboratory parameters, including serum albumin and bilirubin, reached statistical significance, their clinical relevance may be limited given the small magnitude of these differences. These findings suggest that chronological age may reflect increased vulnerability to perioperative stress but does not necessarily preclude safe liver resection. Acceptable short-term outcomes, defined as comparable rates of major postoperative complications and 90-day mortality, can be achieved in carefully selected elderly patients, including octogenarians in contemporary hepatobiliary practice. This interpretation is consistent with recent cohort studies demonstrating comparable perioperative outcomes between elderly and younger patients when patient selection and perioperative management are appropriate [4,5].

### 4.2. Selection Effect and Limitations of Chronological Age as a Risk Indicator

One notable observation in this study is the apparent selection effect among elderly patients. Although age ≥75 years was associated with major postoperative complications in the multivariable model, patients aged ≥80 years did not demonstrate inferior short-term outcomes. However, given the relatively close age ranges and the limited number of events, this finding should be interpreted with caution.

Rather than reflecting a true difference attributable to chronological age alone, this pattern may be influenced by underlying selection processes in clinical practice, whereby patients who undergo hepatectomy at older ages are more likely to have favorable physiological status. At the same time, patients in the 75–79-year range may represent a more heterogeneous group in terms of comorbidity burden and functional reserve.

Consequently, chronological age alone does not fully capture physiologic reserve or perioperative vulnerability. This concept aligns with findings from the broader geriatric surgery literature, where frailty and functional status have been shown to provide prognostic information beyond age alone [6,7,8]. Liver surgery–specific evidence also supports the role of frailty in predicting adverse outcomes after oncologic liver resections [9].

Therefore, the association between age ≥75 years and major morbidity observed in this study should be interpreted with caution and in the context of patient selection rather than as a purely biological effect of chronological age. This association may also be influenced by residual confounding factors that could not be fully accounted for in the multivariable model. In addition, the wide confidence interval observed for this variable likely reflects the relatively limited number of major complication events relative to the number of variables included in the multivariable model, which may have contributed to model instability. Accordingly, this finding should be considered exploratory and interpreted with caution in light of potential model overfitting.

### 4.3. Role of Functional Reserve and Comorbidities in Risk Stratification

In the present analysis, impaired hepatic functional reserve, reflected by a higher ALBI score, and the presence of major comorbidities were independently associated with major postoperative complications. These findings support a perioperative risk stratification strategy that prioritizes liver function and systemic health status rather than chronological age alone.

Previous studies have demonstrated that elderly patients who develop postoperative complications experience disproportionately higher short-term mortality, highlighting the importance of careful patient selection and prevention of severe postoperative morbidity [3]. Current perioperative guidelines also emphasize assessment of frailty and functional status in older adults to improve prediction of postoperative risk and guide perioperative management strategies [6].

In addition, sarcopenia and body composition parameters assessed on preoperative imaging have emerged as reproducible indicators of physiologic reserve and have been associated with adverse postoperative outcomes after liver resection [10]. More recently, multidimensional risk assessment models combining ALBI score with fibrosis-related indices have been proposed to improve prediction of post-hepatectomy liver failure in patients with HCC [14]. Together, these findings highlight the importance of comprehensive physiologic assessment when evaluating elderly candidates for liver resection.

### 4.4. Minimally Invasive Liver Resection in Elderly Patients

A notable characteristic of this cohort was the high utilization of laparoscopic liver resection among elderly patients, particularly among octogenarians. Minimally invasive liver resection has been associated with reduced blood loss, lower perioperative morbidity, and shorter hospital stay compared with open surgery [11]. Evidence focusing on elderly populations suggests that these benefits extend to older patients when procedures are performed in experienced centers [12,15].

Large multicenter propensity score–matched studies have also demonstrated that minimally invasive liver resection can be applied safely in elderly patients with HCC [16,17]. More recent reports suggest that laparoscopic liver resection remains feasible even in octogenarians and in elderly patients with large tumors [18,19]. In the present study, the high rate of laparoscopic surgery likely reflects an institutional strategy aimed at minimizing surgical stress and facilitating postoperative recovery in physiologically vulnerable patients.

In the present study, minimally invasive approaches were limited to laparoscopic hepatectomy, and robotic liver resection was not included. This may represent a limitation, as robotic approaches are increasingly adopted and may offer technical advantages in selected cases. Previous studies have demonstrated that laparoscopic hepatectomy in elderly patients is associated with reduced perioperative morbidity and shorter hospital stay compared with open surgery, without compromising safety [20]. More recent data incorporating robotic approaches further support the feasibility and safety of minimally invasive liver resection, including robotic surgery, in elderly patients [21]. However, the present analysis was not designed to determine the independent effect of minimally invasive surgery on postoperative outcomes. Therefore, these findings should be interpreted within the limitations of the study design.

### 4.5. Technical Considerations and Intraoperative Management

From a technical perspective, efficient surgical conduct, meticulous hemostasis, and appropriate inflow control remain essential considerations during hepatectomy in elderly patients. In the present study, prolonged Pringle maneuver time was independently associated with major postoperative complications. This observation is consistent with previous evidence linking extended inflow occlusion to an increased risk of post-hepatectomy liver failure [22].

At the same time, systematic review evidence suggests that the intermittent Pringle maneuver can reduce intraoperative blood loss without adversely affecting long-term oncologic outcomes when applied appropriately [23]. These findings highlight the need to balance the hemostatic advantages of inflow control with the potential risk of ischemic injury, particularly in patients with limited hepatic reserve.

### 4.6. Study Limitations

The favorable outcomes observed in elderly patients, particularly octogenarians, should be interpreted with caution, as these findings may reflect, at least in part, the selection of patients who met surgical indications and were considered suitable for hepatectomy. The retrospective design introduces the possibility of selection bias, which may be particularly pronounced among very elderly patients who were selected for surgery. In addition, the relatively small number of patients aged ≥80 years limits the ability to detect uncommon adverse events and may reduce the statistical power to identify subtle differences between groups.

However, the present study included only patients selected for surgical resection, and the results should be interpreted within the context of a surgically selected cohort. The absence of objective measures of frailty, sarcopenia, or functional status represents an important limitation of this study. These factors are increasingly recognized as key determinants of postoperative outcomes in elderly patients and may provide more informative risk stratification than chronological age alone. In the present study, surgical candidacy was determined based on clinical evaluation, including hepatic functional reserve and overall operative risk, which may partly reflect the functional status of the patients. Future studies incorporating standardized assessments of frailty and sarcopenia are warranted to better define surgical indications in this population.

Finally, long-term oncologic outcomes were not evaluated because of the limited follow-up duration. Although previous studies have reported comparable oncologic outcomes between elderly and younger patients after curative hepatectomy when appropriate patient selection is applied, the present study was designed to focus primarily on short-term perioperative outcomes.

## 5. Conclusions

Hepatectomy for hepatocellular carcinoma may be performed with acceptable short-term outcomes in carefully selected elderly patients, including octogenarians, in the modern era of minimally invasive liver surgery.

Although advanced age was associated with an increased risk of major postoperative complications in the adjusted analysis, this finding should be interpreted with caution. Chronological age alone should not be considered an absolute contraindication to surgery. Surgical decision-making should instead be guided by comprehensive assessment of hepatic functional reserve, comorbidity burden, and operative risk factors. These findings should be interpreted in light of the limitations of the study.

## Figures and Tables

**Table 1:** Baseline Characteristics of Patients Stratified by Age.

Variables	<80 Years (*n* = 240)	≥80 Years (*n* = 37)	*p*-Value	<75 Years (*n* = 207)	≥75 Years (*n* = 70)	*p*-Value
Sex (female)	49 (20.4%)	14 (37.8%)	0.032	39 (18.8%)	24 (34.3%)	0.012
ASA (III, IV)	159 (66.2%)	21 (56.8%)	0.346	134 (64.7%)	46 (65.7%)	0.997
Previous operation	33 (13.7%)	6 (16.2%)	0.620	28 (13.5%)	11 (15.7%)	0.692
Comorbidity	89 (37.1%)	35 (94.6%)	<0.001	72 (34.8%)	52 (74.3%)	<0.001
ALBI score	3.10 ± 0.41	3.02 ± 0.23	0.217	3.11 ± 0.41	3.03 ± 0.37	0.193
BMI (kg/m^2^)	24.97 ± 3.58	23.93 ± 2.90	0.173	25.05 ± 3.66	24.30 ± 2.95	0.129
MELD score	7.55 ± 1.78	8.41 ± 3.34	0.305	7.47 ± 1.68	8.16 ± 2.64	0.083
Major hepatectomy	160 (66.7%)	20 (54.1%)	0.190	138 (66.7%)	42 (60.0%)	0.387
Laparoscopic resection	194 (80.8%)	35 (94.6%)	0.040	198 (95.7%)	61 (87.1%)	0.337
Tumor Characteristics						
Tumor size (cm)	3.86 ± 2.51	4.92 ± 2.45	0.247	3.78 ± 2.52	4.54 ± 2.47	0.098
Tumor ≥5 cm	63 (26.3%)	14 (37.8%)	0.153	42 (20.3%)	22 (31.4%)	0.081
Multiple tumor	45 (18.8%)	10 (27.0%)	0.249	41 (19.8%)	11 (15.7%)	0.561
Microvascular invasion	65 (27.1%)	9 (24.3%)	0.727	73 (35.3%)	15 (21.4%)	0.045
Preoperative Laboratory Findings						
AST (U/L)	33.5 ± 17.8	29.2 ± 9.9	0.128	34.3 ± 18.1	28.7 ± 9.4	0.180
ALT (U/L)	26.8 ± 18.2	19.3 ± 8.2	0.003	27.7 ± 18.9	20.8 ± 10.7	0.220
Prothrombin Time (%)	90.2 ± 15.7	91.0 ± 14.8	0.811	90.7 ± 15.5	88.6 ± 16.8	0.876
Total Bilirubin (mg/dL)	0.65 ± 0.43	0.55 ± 0.30	0.170	0.64 ± 0.36	0.58 ± 0.34	0.358
AFP (IU/mL)	950.6 ± 2553.7	321.5 ± 417.2	0.322	1022.1 ± 2765.4	441.3 ± 640.6	0.288
PIVKA-II (mAU/mL)	1204.5 ± 2689.5	1411.8 ± 3224.7	0.742	1186.8 ± 2606.9	1364.3 ± 2915.6	0.801

Abbreviations: ASA, American Society of Anesthesiologists physical status classification; ALBI, albumin–bilirubin; MELD, Model for End-Stage Liver Disease; BMI, body mass index; AST, aspartate aminotransferase; ALT, alanine aminotransferase; AFP, alpha-fetoprotein; PIVKA-II, protein induced by vitamin K absence or antagonist-II.

**Table 2:** Operative and Postoperative Outcomes according to Age.

Variables	<80 Years (*n* = 240)	≥80 Years (*n* = 37)	*p*-Value	<75 Years (*n* = 207)	≥75 Years (*n* = 70)	*p*-Value
Intraoperative transfusion	26 (10.8%)	1 (2.7%)	0.146	22 (10.6%)	5 (7.1%)	0.489
Pringle maneuver (cycles)	3.25 ± 1.89	3.18 ± 2.13	0.885	3.31 ± 1.98	3.00 ± 1.55	0.236
Operative time (min)	226.58 ± 74.31	189.65 ± 61.18	0.028	229.09 ± 77.11	203.62 ± 55.36	0.008
Hospital stay (day)	10.53 ± 7.10	13.29 ± 22.19	0.616	10.24 ± 6.90	12.70 ± 14.29	0.241
Estimated blood loss (mL)	537.08 ± 665.83	429.41 ± 256.21	0.163	557.49 ± 707.59	416.00 ± 257.23	0.022
Postoperative AST (U/L)						
POD #1	336.8 ± 296.7	286.5 ± 208.3	0.465	338.2 ± 312.1	314.2 ± 237.6	0.551
POD #3	119.0 ± 110.5	105.9 ± 78.8	0.650	117.2 ± 92.7	122.2 ± 100.6	0.749
POD #5	52.4 ± 32.3	37.5 ± 15.8	0.002	52.1 ± 32.1	49.0 ± 30.1	0.552
Postoperative ALT (U/L)						
POD #1	247.8 ± 229.5	195.2 ± 144.8	0.254	248.9 ± 251.5	225.5 ± 174.2	0.440
POD #3	149.5 ± 137.5	125.4 ± 89.7	0.500	148.5 ± 119.3	145.7 ± 128.4	0.891
POD #5	82.1 ± 71.8	59.8 ± 39.6	0.065	82.4 ± 72.8	73.2 ± 55.3	0.268
Postoperative PT (%)						
POD #1	66.4 ± 17.8	65.4 ± 16.7	0.738	66.9 ± 13.9	63.9 ± 13.7	0.175
POD #3	61.8 ± 16.6	60.5 ± 15.3	0.735	62.1 ± 14.8	59.8 ± 16.0	0.373
POD #5	64.2 ± 15.0	64.2 ± 14.6	0.998	64.0 ± 14.9	63.6 ± 15.2	0.869
Postoperative TB (mg/dL)						
POD #1	1.6 ± 1.4	1.5 ± 1.1	0.707	1.6 ± 0.9	1.6 ± 1.2	0.969
POD #3	1.8 ± 1.5	1.4 ± 0.9	0.195	1.8 ± 1.2	1.7 ± 1.3	0.697
POD #5	1.3 ± 1.1	1.1 ± 0.9	0.424	1.3 ± 1.1	1.3 ± 1.0	0.882
Major morbidity (%)	30 (12.5%)	4 (10.8%)	0.632	20 (9.6%)	14 (20.0%)	0.092
90-day mortality (%)	3 (1.3%)	0 (0.0%)	1.000	4 (1.9%)	0 (0.0%)	0.574

Abbreviations: AST, aspartate aminotransferase; ALT, alanine aminotransferase; POD, postoperative day.

**Table 3:** Multivariable logistic regression analysis of factors associated with major postoperative complications after hepatectomy.

Variables	Odds Ratio	95% CI	*p* Value
Age ≥ 75 years	9.92	2.86–34.45	0.001
Open versus laparoscopic approach	2.34	0.87–6.29	0.091
BMI	0.83	0.71–0.98	0.024
MELD	1.18	0.83–1.66	0.360
ALBI score	6.57	1.17–36.81	0.032
ASA (III/IV)	0.59	0.22–1.60	0.298
Comorbidity	3.85	1.28–11.58	0.017
Underlying Cirrhosis	0.41	0.10–1.75	0.228
Previous operation	1.37	0.27–6.98	0.706
Operative time (per min)	1.00	0.99–1.01	0.418
Estimated blood loss	1.00	0.99–1.01	0.704
Pringle maneuver cylces	1.61	1.16–2.23	0.004
Intraoperative transfusion	1.20	0.32–4.42	0.786

Abbreviations: CI, confidence interval; ALBI, albumin–bilirubin; MELD, Model for End-Stage Liver Disease; ASA, American Society of Anesthesiologists physical status classification; BMI, body mass index. ORs for Pringle maneuver time are expressed per 15 min increase (one clamp cycle).

## Data Availability

The datasets presented in this article are not readily available due to privacy and ethical restrictions.
